# Evaluation of a workplace assessment method designed to improve self-assessment in operative dentistry: a quasi-experiment

**DOI:** 10.1186/s12909-023-04474-z

**Published:** 2023-07-03

**Authors:** Ghaith Alfakhry, Khattab Mustafa, Kamal Ybrode, Bashar Jazayerli, Hussam Milly, Salam Abohajar, Hussam Hassan, Khaled Alhomsi, Issam Jamous

**Affiliations:** 1 Program of Medical Education, Syrian Virtual University, Damascus, Syria; 2 Education Quality and Scientific Research Office, Al-Sham Private University, Baramekeh, City Center, Damascus Governorate, Syria; 3 Faculty of Dental Medicine, Damascus University, Damascus, Syria; 4 Department of Endodontics and Operative Dentistry, Faculty of Dental Medicine, Damascus University, Damascus, Syria; 5 Department of Fixed Prosthodontics, Faculty of Dental Medicine, Damascus University, Damascus, Syria; 6 Department of Periodontology, Faculty of Dental Medicine, Damascus University, Damascus, Syria; 7 Department of Biomedical Sciences, Al-Sham Private University, Damascus, Syria

**Keywords:** Dental education, Self-assessment, Undergraduate, Self-regulated learning, Continuous learning, Workplace assessment, Clinical operative dentistry, Direct observation of procedural skills, Self DOPS, DOPS

## Abstract

**Background:**

Dental education has placed continued emphasis on self-regulated learning (SRL) and its subprocess, self-assessment. This study set out to evaluate the effectiveness of a novel workplace assessment method in developing trainees’ self-assessment of operative procedures.

**Methods:**

A Direct Observation of Procedural Skills (DOPS) form was modified for the use and measurement of self-assessment. Participants were trained on how to conduct self-assessment using the designed assessment form and its grading rubric. Feedback and feedforward sessions were given to address self-assessment and performance issues. A *P-value* less than 0.10 was considered significant and the confidence level was set at 90%.

**Results:**

Thirty-two Year 5 dental students with an age mean of 22.45 (SD = 0.8) completed five self DOPS encounters during the clinical operative dentistry module in 2022. The aggregated total deviation (absolute difference) between self-assessment and teacher assessment decreased consistently in the five assessment encounters with a significant mean difference and a medium effect size (*P* = 0.064, partial Eta squared = 0.069). Participants’ self-assessment accuracy differed from one skill to another and their ability to identify areas of improvement as perceived by teachers improved significantly (*P* = 0.011, partial Eta squared = 0.099). Participants’ attitudes towards the assessment method were positive.

**Conclusions:**

The findings suggest that the self DOPS method was effective in developing participants’ ability to self-assess. Future research should explore the effectiveness of this assessment method in a wider range of clinical procedures.

**Supplementary Information:**

The online version contains supplementary material available at 10.1186/s12909-023-04474-z.

## Introduction

Personal initiative in learning is an important concept that has been given particular emphasis by educational leaders. The Former Secretary of Health, Education, and Welfare in the US stated that “*the ultimate goal of the education system is shift to the individual the burden of pursuing his own education.*” [1]. The previous concept is now popularly known as self-regulated learning (SRL) [2]. Learners with the ability to self-regulate their learning approach educational tasks with confidence, deliberation and resourcefulness; but most importantly, their self-assessment (SA) ability is well-developed [3].

In dental education, SA has been given increasing attention, and in some dental schools, it has been set as a core competence that should be fostered in trainees [4]. Some dental schools teach trainees to self-assess using objective criteria so that they become closer to that of teachers’ assessment (TA) [5]. Nevertheless, the argument about whether SA is a stable characteristic or a learnable skill is still unresolved. In medical education, a longitudinal study detected no significant change in SA over the course of three years [6]. Other studies in dental education replicated this finding [7, 8]. In contrast, there have been some researchers who managed to produce positive findings in developing self-assessment skills in dental education [9]. According to a systematic review, many SA studies in dental education did not report the use of a structured assessment form nor used detailed assessment criteria [10]. The review also highlighted that a number of studies were limited to a single assessment encounter and did not provide any information regarding how students were trained to self-assess [10]. Another shortcoming of SA studies was not exploring trainees’ attitudes [10], which could provide insight into the educational impact of self-assessment, its perceived value, and therefore, students’ motivation to improve this particular skill.

In 2022, a published pilot study investigated whether the SA of trainees can be bridged with TA in clinical operative dentistry by providing trainees with sufficient SA training [11]. This study found a decrease in the gap between SA and teacher assessment (TA) after four assessment encounters. Nevertheless, the previous study was limited by the sample size and the lack of inter-rater and intra-rater reliability statistics. The current study is an extension of the above-mentioned pilot study and is aimed to evaluate the effectiveness of a modified workplace assessment method designed to improve trainees’ self-assessment of clinical performance in operative dentistry.

## Methods

Ethical approval was acquired from the ethical committee at Damascus University Faculty of Dental Medicine on January 15, 2022 (no. 98,735).

### Study design

This is a quasi-experimental study conducted at Damascus University Faculty of Dental Medicine during the second semester of the academic year 2021/2022, which began in March 2022 and ended in June 2022. Participation in this study was voluntary and confidential, and the reported data does not compromise this confidentiality. Participants provided their consent to participate in the recruiting survey. The assessment method used in this study was piloted in 2021 [11].

### Participants and settings

The study was conducted on fifth-year (last year) dental students during the clinical operative dentistry training module in which they had to treat patients in authentic work settings. The required total sample size of participants was calculated using G*Power 3.1 [12] based on the findings of the pilot study [11] for an effect size of 0.64, a power of 90%, and an alpha value of 0.05; the calculated sample size was 28. Participants were recruited in the study via an online survey in which they were also requested to self-evaluate their overall performance in clinical operative dentistry on a 4-point scale similar to that used in the study.

To ensure fairness between participants and non-participants, it was explicitly stated that partaking in the study would not affect their grades in the module which would be assigned based on the traditional assessment criteria of the Faculty as their non-participating peers.

### Assessment method

Participants were assessed using the DOPS (Direct Observation of Procedural Skills) method and were instructed to perform self DOPS. In comparison to the pilot study [11], the grading scale was reduced from a 5-point scale to a 4-point scale (1 = clear fail, 2 = borderline fail, 3 = borderline pass, 4 = clear pass) because participants rarely hit the excellent point; second, two assessment criteria namely *tooth preparation* and *restoration* were subdivided into 4 and 7 sub-criteria respectively (supplementary file I). The English version is available in supplementary file I. The DOPS forms for clinical teachers and participants were identical except for the addition of item coded III (supplementary file I) to the clinical supervisor’s form which was designed to assess participants’ abilities to pinpoint areas of improvement and excellence. All forms designed and used in the study were in Arabic to overcome the language barrier. A grading rubric was also shared with both participants and supervisors; the rubric provided a detailed description of each intersection between a scale point and a criterion (supplementary file II). All documents went through a double-translation process to ensure the accuracy of the language.

Participants underwent five DOPS assessment encounters in which they assessed their own performance and were also assessed by a clinical supervisor simultaneously. Between each encounter, there was a one-week interval. Participants independently completed the form directly after they finished their procedure (retrospectively), whereas supervisors completed the form directly as participants were conducting the procedure. Participants did not receive feedback from supervisors until they have completed the self-assessment form.

There were three calibrated clinical assessors. Each student was assessed at least once by each supervisor. This was done to increase assessment inter-reliability [13] and mitigate possible bias that might result from participants becoming calibrated to a certain supervisor.

Five assessment encounters were decided to be the minimum number of encounters required by each participant; a reliability study of DOPS found that five encounters achieved a generalizability coefficient of 0.87 (95% CI: 0.59) [14] despite the fact that assessors were not trained. The previous pilot study [11] conducted four encounters and found significant findings; hence, five encounters were set to be sufficient, especially since the assessors were calibrated; further assessment encounters were difficult to achieve due to pragmatic reasons and limited human resources. Data from those who completed less than 5 encounters was excluded from the analysis.

### Calibration of clinical supervisors

Three qualified GP dentists were chosen as clinical supervisors. These clinical supervisors had experience in conducting DOPS assessments as they participated as assessors in the pilot study. Moreover, the grading rubric was discussed in detail between the three supervisors to make sure that all agreed upon the meaning of each criterion.

Prior to commencing the study, the inter-rater reliability of the three clinical supervisors was evaluated on three DOPS occasions in which they assessed two different participants conducting three different classical operative procedures on real patients; one participant had done one procedure and the other had done two procedures. This evaluation procedure assessed inter-rater reliability across different cases.

As for the intra-rater reliability, a simulated trainee-scenario paper-based exam was designed. The rationale behind using the simulated scenario is that it can be repeated exactly the same, whereas a participant’s clinical performance can be inconsistent on different occasions. Supervisors had to evaluate the simulated trainee’s scenario using the DOPS form twice with a one-week interval. Thereafter, the intra-rater reliability was calculated for each supervisor.

During the actual DOPS encounters, the clinical supervisors did not intervene unless the participant was totally incapable of completing a certain step of the procedure, and in this case, only, the supervisor assigned the performing participant the lowest grade on that certain skill before taking over the participant and completing the step.

### Self-assessment training

A full detailed description of self DOPS assessment protocol was sent to participants along with the DOPS form and grading rubric; all sent documents were in Arabic. An instructional video that explains the assessment process and criteria was filmed and sent to participants; the instructional video demonstrated correct application of the grading rubric and assessment form on scenario cases. These scenario cases covered cases that were *clear pass, clear fail* as well as *borderline fail/pass*. Before the clinical assessment encounters and to ensure that participants read and understood the assessment instructions, an electronic quiz was made in which a virtual case was put forward to participants to assess using the DOPS form and grading rubric. After submitting their answers to the quiz, participants could assess the discrepancy in their evaluations compared to the actual evaluations assigned by the clinical supervisors. Further, in line with training students to self-assess, the grading criteria, assessment approach, and basic assessment skills were illustrated to participants face-to-face before the study commenced.

Before each self DOPS encounter, clinical supervisors held a 5-minute feedforward session with participants to discuss the pitfalls they noticed participants making in assessing themselves according to the observations of the previous session [15]. After each Self DOPS, a 5-minute feedback session took place during which clinical supervisors compared their scores with that of the self-assigned ones and thereafter discussed the differences in scoring with participants so that the reason and/or the specific observations that merited a certain score were clear to participants. Further, areas of improvement and excellence were highlighted and an action plan to improve clinical performance was agreed upon. This model of starting a feedback discussion with self-assessment has been widely supported [16–18], and it is based on multiple pedagogical theories suggesting that feedback providers should engage in a dialogue with feedback receivers; self-assessment being a good conversation starter [19]. This sequence is especially useful in providing negative feedback as it is easier for faculty to ask participants about what they did wrong and then elaborate in comparison to directly criticizing their performance; the former approach is less pejorative [20]. Further, utilizing a standard assessment form for both participants and supervisors helps in making the content of the feedback provided by both sides closely related; an aspect that previous studies were limited by [20].

### Quantifying self-assessment accuracy

Three main methods were used to quantify self-assessment accuracy. First, the mean arithmetic difference between SA and TA scores indicated how much participants overestimated or underestimated their performance at each item. Second, the sum of deviations (absolute differences) between SA and TA scores of each of the 22 items indicated how far SA was from that of TA. The deviation was calculated at the domain level as well. These two methods of quantifying self-assessment accuracy were inspired by a previous study [6].

The third method of quantifying self-assessment accuracy is novel to our study and was illustrated in the pilot study [11]; briefly, on the back page of the DOPS form (supplementary file I), participants and supervisors were asked to separately identify three areas of improvement and three areas of excellence. Thereafter, the clinical supervisors compared their points to that of participants and assessed how many points identified by participants matched theirs on a 4-point scale (0 = no matching points, 1 = one matching point, 2 = two matching points, 3 = three matching points). This variable assessed participants’ ability to identify the most serious areas of improvement and the most prominent areas of excellence in their own performance as perceived by the more experienced and calibrated clinical supervisors.

To examine changes in participants’ performance, the sum of points given by the supervisors for each participant (at each of the 22 items) was calculated and the mean was used as the main indicator of participants’ performance.

### Participant’s attitudes towards the assessment method

Participants’ attitudes toward the self-assessment method were assessed after each encounter on the DOPS assessment form in two items (a- and b-, supplementary file I, pg.4).

### Data analysis

The skewness of data and histograms were used to examine data normality. Minor violations of normality were disregarded as the sample size is over 15 (sample size = 32) [21]. The intraclass correlation coefficient was used to measure inter-rater and intra-rater reliability. Paired *t*-test was used to measure the mean difference between SA and TA at each item across different encounters. Deviation (absolute difference) between SA and TA scores was calculated for each domain at each encounter. Repeated-measures ANOVA was used to measure the difference between deviation mean scores of the five encounters; a *P*-value < 0.10 was considered significant and the confidence level was set at 90% in all conducted statistical tests as per previous recommendations [22]. The significant test was made less stringent considering the human factor as well as the limited assessment encounters. Statistics conducted did not focus merely on hypothesis testing but also estimation; therefore, effect size and observed power were reported whenever possible. Mixed ANOVA was used to test for the effect of sex, percentage grade (PG), case complexity and restoration type on the deviation mean scores in the five encounters. Friedman’s two-way analysis was conducted to measure the difference in the number of matching points between participants and supervisors in regard to areas of improvement and areas of excellence.

Data processing and analysis were conducted using Microsoft Excel (2019) [23] and IBM SPSS Statistics for Windows, version 26 (IBM Corp., Armonk, N.Y., USA). G*Power 3.1 [24] was used to calculate the sample size. Google Forms [25] was used to conduct the screening survey.

## Results

Out of 182 trainees, 87 trainees completed the recruiting survey and only 39 trainees were selected for the study based on who completed the survey first. Seven trainees did not manage to complete all five required assessment encounters and therefore were excluded from the analysis. A total of 32 participants met the inclusion requirement. The age mean for the participants was 22.45 (SD = 0.8) and consisted of 25% (n = 8) male participants. As for their reported percentage grade (PG), 6.3% (n = 2) had a PG of 70–75%, 28.1% (n = 9) had a PG of 75–80%, 53.1% (n = 17) had a PG of 80–85% and 12.5% (n = 4) had a PG of 85–90%. The majority of cases (82.5%) treated by participants were resin composite restorations; the total number of cases was 160 (32 participants * 5 encounters). Table 1 provides a summary of the cases participants treated during the 5 assessment encounters. The inter-rater reliability as measured by the intraclass correlation coefficients for the three supervisors in the first, second, and third pre-study encounters were 0.746 (*P* = 0.004), 0.794 (*P* < 0.001), and 0.790 (*P* < 0.001) respectively. As for the intra-rater reliability, the intraclass correlation coefficients for the three supervisors were 0.982 (*P* < 0.001), 0.713(*P* = 0.002), and 0.843 (*P* < 0.001).

**Table 1:** Summary of assigned supervisor, case complexity, restoration material and restoration class of participants’ cases in the five occasions in total (n = 160)

Case complexity	19.4% (missing)	28.7% (easy)	36.3% (average)	14.4% (complex)			
Restoration material	3.8% (missing)	82.5% (resin composite)	10.0% (amalgam)	3.8% (GIC)			
Restoration class	2.5% (missing)	12.5% (class I)	30.0% (class II)	15.6% (class III)	19.4% (class IV)	11.3% (class V)	8.8% (extensive restoration)

GIC: Glass ionomer cement

The changes over time in the mean differences (MDs) between SA and TA scores (SA-TA) at each assessment criterion are illustrated in Table 2. Most MDs were positive and this indicated that SA scores were higher than TA. As for the changes across different encounters, MDs of most items decreased through the five encounters. The number of items with a statistically significant difference (*P* < 0.10) between TA and SA was 17, 10, 11, 7, and 10 in the first, second, third, fourth, and fifth encounters respectively. It is also important to note that *P*-value increased across encounters from mostly < 0.001 to higher values as shown in Table 2. In items no. 18 and 20, the MDs were consistently negative indicating that TA scores were higher than SA. In items no. 11, 14, and 15, MDs were initially positive but changed to negative later in the 4th and 5th encounters. MDs between SA and TA remained statistically significant across the five encounters in items no. 1, 2, 3, 6, and 7; nevertheless, the *P*-vale decreased as shown in Table 2. The MD of the *Overall performance assessment* (item no. 22) dropped from 0.77 ± 0.7 in the first encounter with a significant difference (*P* < 0.001) to 0.29 ± 1.0 in the 5th encounter with no significant difference between SA and TA (*P* > 0.10).

**Table 2:** Paired *t*-test showing the mean difference between self-assigned scores and that of clinical assessors at each assessment criteria in each encounter

	1st encounter	2nd encounter	3rd encounter	4th encounter	5th encounter	Total
Criteria	MD ± SD	MD ± SD	MD ± SD	MD ± SD	MD ± SD	MD ± SD
***Clinical knowledge and judgment***						
1. Clinical examination, diagnosis and treatment planning	0.96 ± 1.0***	0.46 ± 1.1*	0.75 ± 0.6***	0.53 ± 1.0**	0.48 ± 1.0*	0.63 ± 0.9***
2. Demonstrates understanding of indications, dental materials and used technique	0.85 ± 1.1***	0.07 ± 1.1	0.71 ± 0.9***	0.46 ± 0.9*	0.66 ± 1.0**	0.55 ± 1.0***
***Professionalism, patient management and ergonomics***						
3. Obtaining patient consent after explaining the procedure and possible complications	1.60 ± 0.8***	0.70 ± 1.3*	1.00 ± 0.9***	0.80 ± 1.0***	0.89 ± 1.2**	1.02 ± 1.1***
4. Pre-procedural preparation	0.59 ± 0.9**	0.32 ± 1.2	0.67 ± 0.8***	0.37 ± 1.0*	0.45 ± 1.2	0.48 ± 1.0***
5. Infection control	0.65 ± 0.8***	0.31 ± 0.9*	0.12 ± 0.8	0.37 ± 1.1*	0.40 ± 1.1*	0.37 ± 0.9***
6. Pain, anxiety management	0.93 ± 1.2***	0.42 ± 1.0*	0.83 ± 0.8***	0.46 ± 1.1*	0.69 ± 1.0**	0.67 ± 1.0***
7. Communication skills with patient and team	1.15 ± 1.0***	0.56 ± 1.2*	0.41 ± 1.0*	0.46 ± 1.2*	0.70 ± 0.9***	0.66 ± 1.1***
8. Patient education	0.42 ± 0.9*	0.41 ± 1.1*	0.52 ± 1.1*	0.17 ± 0.8	0.36 ± 1.1	0.38 ± 1.0***
9. Time management	0.22 ± 0.9	0.15 ± 0.9	0.51 ± 1.0*	0.03 ± 1.0	0.16 ± 1.2	0.21 ± 1.0*
10. Ergonomics	0.53 ± 0.7***	0.09 ± 1.3	-0.03 ± 0.9	0.10 ± 0.9	0.31 ± 0.8*	0.20 ± 0.9*
***Tooth preparation***						
11. Isolation	0.33 ± 0.8*	0.24 ± 0.9	0.53 ± 0.9**	-0.03 ± 0.8	-0.38 ± 1.0*	0.13 ± 0.9
12. Initial and final access (over-/under-extension/adjacent tooth damage)	0.52 ± 1.0*	0.26 ± 1.1	0.28 ± 1.0	0.06 ± 1.1	0.06 ± 1.1	0.22 ± 1.0*
13. Caries removal	1.00 ± 0.9***	0.46 ± 0.9*	0.58 ± 0.8**	0.20 ± 1.0	0.14 ± 1.0	0.46 ± 1.0***
14. Unsupported enamel removal	0.25 ± 0.9	0.06 ± 1.1	-0.16 ± 1.3	-0.33 ± 1.0	-0.13 ± 0.9	-0.05 ± 1.1
***Tooth restoration***						
15. wedging and matrix placement	0.33 ± 1.0*	-0.17 ± 1.2	0.12 ± 0.9	-0.33 ± 1.1	-0.15 ± 0.9	-0.03 ± 1.1
16. Etching and bonding (Composite)	0.53 ± 0.7**	0.30 ± 1.0	0.40 ± 1.0	0.00 ± 0.7 (*P > 0.99*)	-0.11 ± 0.9	0.24 ± 0.9**
17. Cavosurface (excess/submargination)	0.58 ± 0.8**	0.12 ± 1.0	0.18 ± 0.7	0.03 ± 0.7	0.12 ± 1.0	0.20 ± 0.9**
18. Color matching and/or surface polishing	-0.19 ± 1.1	-0.26 ± 1.1	0.10 ± 0.8	-0.34 ± 1.1	-0.40 ± 1.0*	-0.22 ± 1.0*
19. Axial anatomy (buccal, lingual, proximal, contact point)	0.22 ± 1.0	-0.21 ± 1.1*	-0.27 ± 1.1	0.03 ± 1.2	0.03 ± 0.8	-0.03 ± 1.0
20. Occlusal/Incisal edge anatomy (not to be evaluated in class III or V)	0.00 ± 1.3 (*P* > 0.99)	-0.77 ± 1.5*	-0.40 ± 1.0*	-0.20 ± 0.9	-0.38 ± 1.0*	-0.34 ± 1.1**
21. Occlusion	0.80 ± 1.1**	0.09 ± 1.5	0.00 ± 0.8 (*P > 0.99*)	-0.14 ± 0.8	0.00 ± 1.2 (*P* > 0.99)	0.16 ± 1.1
***22. Overall performance assessment***	0.77 ± 0.7***	0.31 ± 0.9*	0.25 ± 1.3	0.22 ± 0.88	0.29 ± 1.0	0.36 ± 1.0***

MD: Mean difference, SD: Standard deviation which is at a 9*0*% confidence interval. Positive values indicate that trainees’ scores are higher than that of assessors and negative values indicate the opposite. *p < 0.10, **p < 0.01, ***p < 0.001

The deviations (absolute differences) between SA and TA in each domain across the five encounters are demonstrated in Table 3. Deviation in all domains except for *tooth preparation* decreased in the last two encounters in comparison to the first two encounters. Deviation of *overall performance assessment* (item no. 22) also decreased slightly. The total deviation of all 22 items dropped consistently across encounters (Table 3). A repeated-measures ANOVA, with sphericity assumed, was conducted to assess the difference between the deviation scores of the five encounters. The results of the repeated-measures ANOVA are illustrated in Table 3. The *aggregated total deviation* mean differed significantly across the five encounters, *P* = 0.064, with a medium effect size and an observed power of 65.1%. Polynomial contrasts indicated, that there was a statistically significant linear trend in the changes of the aggregated total deviation across encounters, *F*(1, 31) = 5.659, *P* = 0.024 with a large effect size, partial Eta^2^ = 0.154 and an observed power of 0.752. There was a significant difference in the deviation score of the *tooth restoration* domain, *P* = 0.068, partial Eta squared = 0.068. The largest *P*-value and the smallest effect size in the assessment domains were observed in the *tooth preparation* domain, *P* = 0.874, partial Eta squared = 0.010. In terms of changes in TA across the five encounters, the sum of points for all 22 items given by supervisors fluctuated between encounters with a rising tendency (Table 3). A repeated measures ANOVA was conducted to assess whether there were differences in TA between the five assessment encounters. Results indicated that teachers’ rated participants’ performance significantly differed across encounters, (*F* = 3.986, *P* = 0.011, partial Eta^2^ = 0.363). Bonferroni multiple comparisons detected a significant difference (*P* = 0.005) between TA in the first and fourth encounters. The sum of SA points for the 22 items is illustrated in Table 3. SA mean scores fluctuated slightly with a decreasing tendency. Repeated-measures ANOVA did not indicate a significant difference between SA of the five encounters, *P* = 0.539 with slightly over small effect size, partial Eta^2^ = 0.025, and an observed power of 0.361. At the assessment criteria level (considered separately), it was observed that SA mean scores generally declined after each encounter, whereas TA scores increased (Supplementary file III).

**Table 3:** Absolute differences between self-assigned scores and that of clinical assessors in each assessment domain with repeated measures ANOVA statistics

	1st encounter (MD ± SD)	2nd encounter (MD ± SD)	3rd encounter (MD ± SD)	4th encounter (MD ± SD)	5th encounter (MD ± SD)	Repeated measures ANOVA^2^			
						*F*	*P*	Partial Eta squared	Observed power^3^
Clinical knowledge and judgement	1.97 ± 1.4	1.56 ± 1.2	1.59 ± 1.0	1.50 ± 1.3	1.63 ± 1.0	0.877	0.480	0.028	0.393
Professionalism, patient management and ergonomics	7.47 ± 2.8	6.56 ± 3.6	6.28 ± 2.8	6.22 ± 2.7	5.94 ± 3.0	1.553	0.191	0.048	0.599
Tooth preparation	2.69 ± 1.1	3.03 ± 1.9	2.59 ± 1.8	2.81 ± 1.5	2.84 ± 1.6	0.305	0.874	0.010	0.198
Tooth restoration	5.00 ± 2.4	5.03 ± 2.7	4.06 ± 2.3	4.16 ± 2.3	3.59 ± 2.3	2.256	0.067*	0.068	0.758
Overall performance assessment	0.90 ± 0.5	0.75 ± 0.67	0.93 ± 0.8	0.68 ± 0.5	0.74 ± 0.7	1.086	0.368	0.042	0.459
Aggregated Total deviation^1^	18.0 ± 5.4	16.9 ± 7.4	15.3 ± 5.1	15.3 ± 4.6	14.7 ± 5.1	2.282	0.064*	0.069	0.763
Aggregated Self-assessment scores	63.34 ± 10.39	61.12 ± 10.56	64.06 ± 9.77	61.93 ± 8.69	61.09 ± 10.80	0.782	0.539	0.025	0.361
Aggregated Teacher assessment scores	47.18 ± 8.05	53.18 ± 9.20	51.40 ± 8.92	54.87 ± 11.38	49.84 ± 11.52	3.411	0.011	0.099	0.907

^1^ The sum of absolute differences between self-assessment and assessors’ scores for all of the 22 items. The maximum aggregated score a participant can get is 88 (22 items * 4 points)

^2^ Sphericity assumption was met and assumed

^3^ observed power was computed using an alpha value of 0.10

* significant difference *P* < 0.10

Mixed ANOVA showed no statistically significant effect for sex, age, and percentage grade on the aggregated total deviation score, *P* > 0.10 (Table 4). Similarly, one-way ANOVA showed no significant effect, *P* > 0.10, for case complexity, restoration material, and restoration type on the aggregated total deviation mean score with a small effect size. Only age had a large effect size (partial eta^2^ = 0.202) but with no statistically significant effect (*P* = 0.194) (Table 4).

**Table 4:** Mixed ANOVA statistics showing the effect of sex, age, percentage grade and case-related factors on the aggregated deviation score across each of the five assessment encounters

	Mixed ANOVA		
	*F*	*P*-value	Partial Eta squared
Sex	0.064	0.803	0.002
Age	1.641	0.194	0.202
Percentage grade	0.081	0.970	0.009
	**One-way ANOVA**		
	***F***	***P*** **-value**	**Eta squared**
Case complexity	0.775	0.569	0.025
Restoration material	0.286	0.835	0.005
Restoration class	0.744	0.615	0.028

**Fig. 1:**
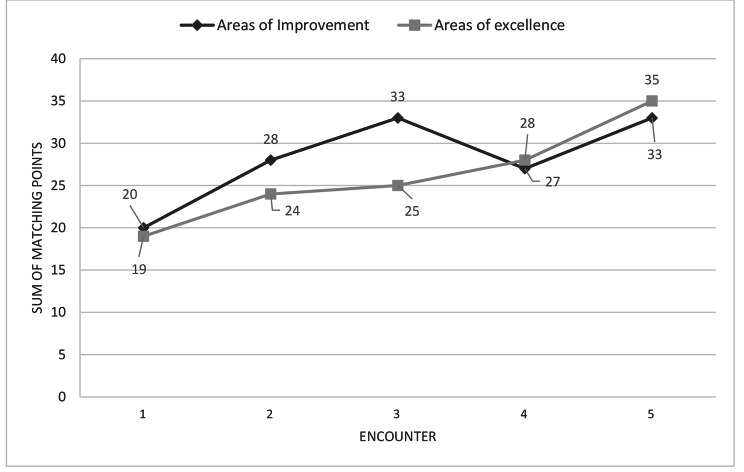
Changes in sum of matching points between participants and assessors in the areas of improvement and areas of excellence across the five encounters

Figure 1 shows the sum of matching points between participants and supervisors in areas of improvement and areas of excellence in each of the five encounters. The number of matching points of areas of improvement between participants and supervisors increased significantly according to Friedman’s two-way analysis (*P* = 0.044) from only 20 matching points in the first encounter to 33 matching points in the fifth encounter. Similarly, the number of matching points for areas of excellence between participants and supervisors also increased consistently and significantly (*P* = 0.042) across the five encounters.

**Fig. 2:**
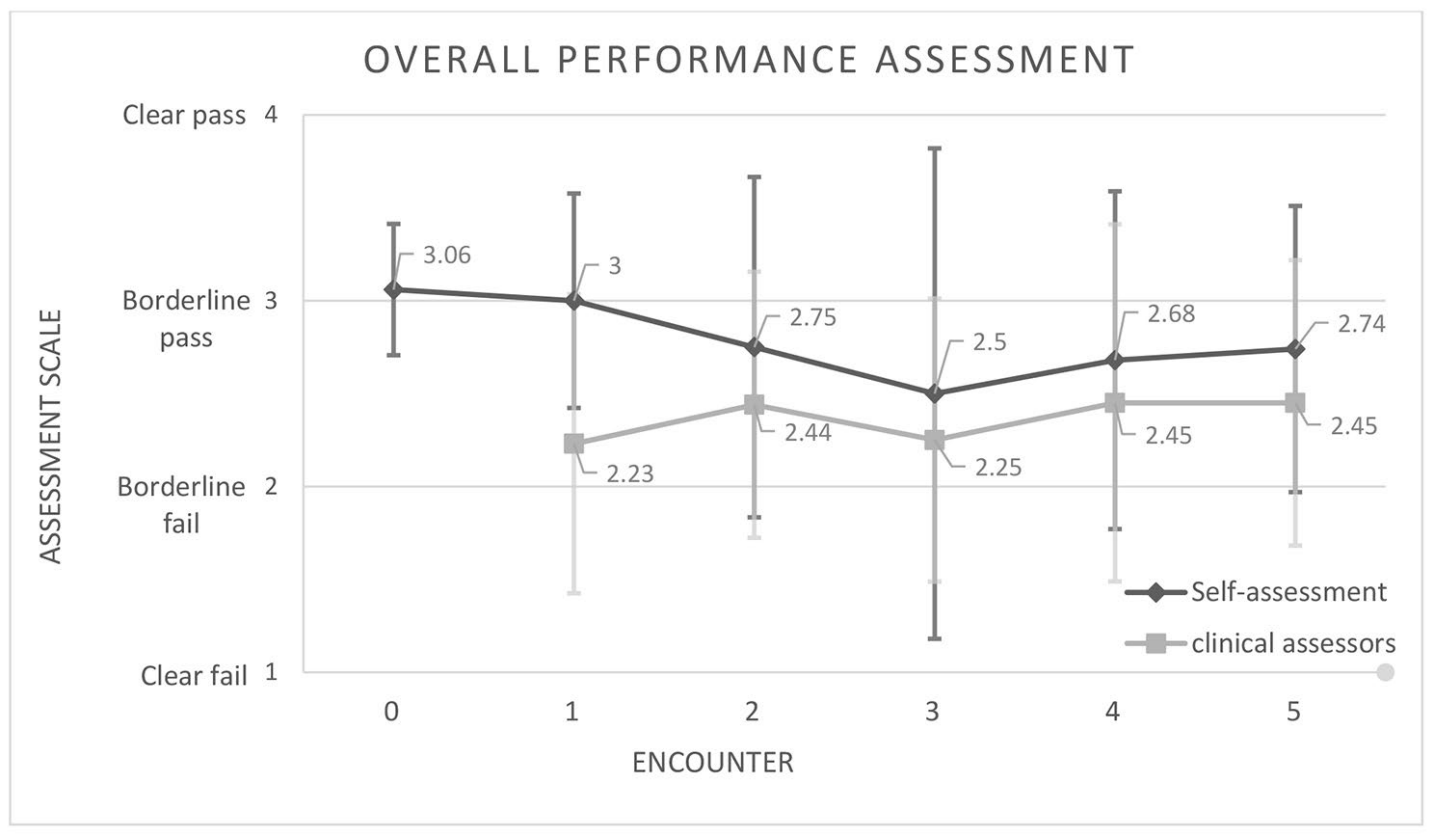
Mean of participants’ self-assigned scores and that of clinical assessors in the overall performance assessment item (n.22) in the screening survey (encounter 0) and the in the 5 DOPS encounters

SA and TA of overall performance assessment (item no. 22) in the screening survey and each of the five encounters are demonstrated in Fig. 2. The figure shows that the SA of overall performance in the screening survey and the first DOPS encounter were quite similar and the paired-sample t-test showed no significant difference (MD = 0.06, SD = 0.56, 90% Confidence Interval: -0.10 to 0.23, *P* = 0.536). In the following encounters, self-assessment started to follow in trend with that of TA, increasing when it increased and decreasing when it decreased.

### Participants’ attitudes towards the assessment method

After each DOPS encounter participants had to indicate their disagreement or agreement on a 4-point scale with two statements that examine participants’ attitudes (a- and b-, supplementary file I, pg.4). The first statement was *Your experience with the current assessment method was positive* which all participants across all encounters agreed with; the exception for this was one participant who strongly disagreed with the statement in two out of the five DOPS encounters. The second statement being *You benefited from participating and evaluating yourself with the supervisor*, which all participants agreed with across all five encounters.

## Discussion

This study was set out to evaluate the efficacy of a modified workplace assessment method (self DOPS) designed to improve trainees’ self-assessment of clinical performance in operative dentistry. To this end, a quasi-experiment was conducted on 32 participants who engaged with self DOPS as well as were evaluated by trained and calibrated clinical supervisors across five encounters. The inter- and intra-rater reliability statistics indicated good reliability of these supervisors.The findings of this study showed that SA was consistently higher than TA across encounters; nevertheless, the aggregated total deviation (absolute difference) between SA and TA decreased considerably over the course of five DOPS encounters. The gap between SA and TA differed from one skill to another. SA and TA both fluctuated between encounters, but in general, SA decreased and TA increased. Participants’ ability to identify areas of improvement and areas of excellence consistently improved over the five encounters. Similarly, participants’ clinical performance as assessed by supervisors improved steadily in the first four encounters yet dipped in the last encounter. Positive attitudes towards the assessment method utility were expressed by participants.

According to the triangulation of different measures of self-assessment, the introduced assessment method seemed effective in improving participants’ self-assessment ability in operative dental procedures, albeit SA accuracy varied from one assessment criterion to another. In dental education, there have been several attempts to improve trainees’ ability to self-assess; some were successful [7, 9, 26–28], while others were not [7, 29]. Common features of successful approaches to improving SA were providing focused training and feedback as well as using clear objective criteria and allowing students enough time to develop this skill [9, 26–28]. Repetitive experience with SA in conjunction with corrective feedback might also be a contributing factor in improving SA accuracy [30]. A recent study that utilized digital assessment to improve SA accuracy of dental anatomy wax-up exercise reported positive findings; this study provided participants with clear evaluation criteria, a lecture on conducting SA, and practice sessions where students compared their scores with that of instructors along with access to the evaluation software for feedback [9]. Most of these elements are similar to the self-assessment experience participants were subjected to in the current study. This comparison with previous studies shed light on the elements of the assessment method used in this study that is believed to have affected SA the most, which were: the use of clear assessment criteria, focused SA training, repetitive experience with SA accompanied with corrective TA. Moreover, unlike some assessment models that rely only on feedback, the proposed assessment model introduced feedforward sessions in which assessment criteria were discussed in detail with students to calibrate and guide self-assessment practice in the immediate future encounter [15]. This may have helped participants understand the assessment criteria and therefore self-assess more accurately.

According to a systematic review in 2016 on self-assessment in dental education [10], the majority of the literature in dental education did not report the use of a structured self-assessment training. In contrast, the current study reports in detail structured and various approaches to familiarize and train participants with self-assessment such as using instructional video, orientation session, electronic quiz as well as feedforward and feedback before and after each assessment encounter; all of these approaches were not difficult to implement and could be feasible at a larger scale. As for faculty calibration, very limited studies in the literature reported the amount and approach to faculty calibration [10], and, to our knowledge, few studies reported inter-rater or intra-rater reliability [8, 10, 31]. This adds to the significance of the current study which reported both inter-rater and intra-rater reliability and calibration method.

Self-assessment has been previously described as a stable characteristic that changes little over time [6]. This can be true if no repetitive correction of SA took place as illustrated in this study where SA mean score of overall performance in the screening survey with no corrective feedback and in the first encounter were approximately equal (Fig. 2). The aggregated SA mean scores of the five encounters were not significantly different. Nevertheless, that does not necessarily mean that SA did not change; it is important to note that SA scores did not rise even though teachers were giving students better scores after each encounter but it rather decreased overall. It is possible that a significant difference in SA could have been detected if more assessment encounters were conducted. Further evidence of improvement in the SA ability of participants are present in this study such as: (1) the reduced gap between SA and TA encounter after encounter, (2) improvement in participants ability to pinpoint areas of improvement and areas of excellence.

Participants overcalled their performance in the majority of assessment criteria and this is concurrent with other study findings [10, 11]. This might reflect the lack of experience in performing operative dental procedures [7, 32], or might show that participants were injecting their emotions consciously or subconsciously during the assessment process as a defense mechanism.

In this study, SA of technical skills (tooth preparation and tooth restoration) was closer to that of TA in comparison to non-technical skills (knowledge and professionalism). This is concurrent with previous findings in surgical and dental training [11, 33]. Judging one’s performance is relatively straightforward when it produces simple objective outcomes such as avoiding excess or sub-margination (item no. 17) or achieving optimal tooth anatomy (no. 19, 20) [3]. On the other hand, assessing covert interpersonal skills such as professionalism and patient management skills have varying subjective outcomes, making the assessment process more difficult.

**Fig. 3:**
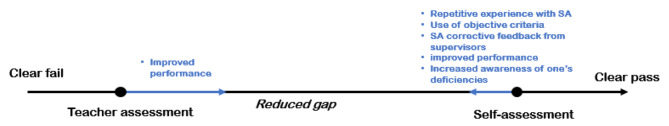
A model that highlights the factors leading to the reduced gap between TA and SA in the current study

It is out of the scope of this study to determine the relationship between conducting self-assessment and performance levels. Nevertheless, participants’ scores as given by teachers consistently improved over the course of four encounters and dropped slightly in the fifth encounter. The dip in participants’ performance in the fifth encounter might be because it is the last session participants have to finish their assigned clinical cases before the end of the term. In a former study in a removable prosthodontics lab, self-assessment and reflection were found beneficial in improving future performance [34]. Zimmerman’s SRL cyclical model shows how self-assessment and performance are mutually dependent. For instance, self-assessment can help an individual identify learning goals and increase one’s motivation which in turn can affect performance [3, 35]. It’s only logical for self-assessment to change according to performance. The skills needed to perform the metacognitive task of SA are exactly the same as the cognitive tasks necessary to perform well at a certain procedure [36]. Similarly, performance improvement can also affect SA in what is popularly known as the Dunning–Kruger Effect [37]. This could explain why our novice participants’ inflated SA scores at the first encounter declined when performance improved. A model that illustrates the factors leading to a reduced gap between SA and TA is illustrated in Fig. 3.

The complexity of the clinical environment makes it difficult to know for sure whether the introduced assessment method or some other variable affected SA accuracy. For example, the patient and the dental assistant’s role were not taken into consideration in the current study. Task familiarity is another factor that can affect participants’ ability to self-assess; this limits the transferability of our findings to other procedures. Although many variables such as time, case difficulty, and procedure were taken into consideration, this study’s results need to be interpreted with an understanding of the nature of the complex clinical environment.

Implementation of DOPS as proposed in the protocol of this study should have similar feasibility to the regular DOPS with no self-assessed forms. The long amount of time the assessor has to spend with the assessee is a feasibility issue that was reported in the previous literature and were faced in this study [14]. This issue should be especially problematic when shortage of staff and large student count are apparent as in the case Damascus University dental school [38, 39]. Nonetheless, adding the self-assessment component to DOPS did not increase the usual amount of time taken for feedback despite the focus on fostering on self-assessment. In actuality, it made the feedback session more focused and efficient as the teacher skipped discussing the points in which the trainee showed confirming self-assessment and focused primarily on aspects that have disconfirming self-assessment. The proposed DOPS model adopted in this study should be as pedagogically effective as regular DOPS [40] and it could be more student-centered thanks to its self-assessment component; this might increase acceptability of DOPS among students. To allow the implementation of this self-assessment focused DOPS at a larger scale at dental schools such as Damascus University Faculty of Dental Medicine, it is suggested that the university should reform its assessment policy to allow the adoption and experimentation with for-learning assessment methods; second, the university should increase funding to train the clinical staff; third, the university should modify its admission policy taking into consideration the staff capacity [38, 39].

Despite its limitations, the current study findings add to the body of the literature concerned with improving the SA accuracy of dental trainees. Few studies have tried to improve SA in authentic clinical work settings [10]; this makes the current study an important addition, especially with the results it held as to the improvement in SA ability of participants. It is well-established in the scientific community that students have poor self-assessment abilities [41, 42] and evidence did not suggest the improvement of this skill without intervention over time [6, 43]. This urges for the exploration of novel structured methods to teach students how to accurately self-assess their performance in the hectic clinical environment [10]. It would be of interest for future research to explore how this self-DOPS affects students’ SRL processes.

## Conclusion

The findings of this study suggest that the introduced assessment method was effective in fostering participants’ ability to self-assess and identify areas of improvement in the clinical environment. A natural progression of this work is to evaluate the effectiveness of the introduced assessment method in developing participants’ self-assessment of performance in a wider range of clinical procedures.

## Electronic supplementary material

Below is the link to the electronic supplementary material.

Supplementary Material 1

Supplementary Material 2

Supplementary Material 3

## Data Availability

The datasets used and/or analysed during the current study are available from the corresponding author upon reasonable request.
