# Supplementary material for: Evaluation of a workplace assessment method designed to improve self-assessment in operative dentistry: a quasi-experiment
Source: BMC Med Educ. 2023 Jul 3;23:491. doi: 10.1186/s12909-023-04474-z (PMC10318704; doi:10.1186/s12909-023-04474-z)
Supplement: Supplementary file 1 — Supplementary Material 1 [file 12909_2023_4474_MOESM1_ESM.docx]

**DOPS Form for Clinical Teachers**

Date: ……/……/2022 Supervisor’s name:. ……….............. Trainee’s name: ………………..
Procedure: Amalgam/Composite/GIC Class: I / II / III / IV / V / 6 Case complexity: Easy/Moderate/Complex
Observation duration:……... minute Evaluate the trainee’s performance according to the following scale:

| Scale | 1. Clear fail | 2. Borderline fail | 3. Borderline pass | 4. Clear Pass | 0. Unable to comment |
| --- | --- | --- | --- | --- | --- |

| Skill | Score | Note |
| --- | --- | --- |
| Clinical Knowledge and Judgment | | |
| 1. Clinical assessment, diagnosis and treatment plan |  |  |
| 2. Demonstrates understanding of indications, dental materials and used technique |  |  |
| Professionalism, Patient management and Ergonomics | | |
| 3. Obtaining patient consent after explaining the procedure and possible complications |  |  |
| 4. Pre-procedural preparation |  |  |
| 5. Infection control |  |  |
| 6. Pain and anxiety management |  |  |
| 7. Communication skills with patient and team |  |  |
| 8. Patient education |  |  |
| 9. Time management |  |  |
| 10. Ergonomics |  |  |
| Tooth Preparation | | |
| 11. Isolation |  |  |
| 12. Initial and Final access (*over or under-extended, adjacent tooth damage)* |  |  |
| 13. Caries removal |  |  |
| 14. Unsupported enamel removal |  |  |
| Tooth Restoration | | |
| 15. Matrix placement and Wedging |  |  |
| 16. Etching and bonding |  |  |
| 17. Cavosurface (Excess/Submargination) |  |  |
| 18. Restoration Surface/Shade |  |  |
| 19. Axial Anatomy (Facial, lingual, proximal contours) |  |  |
| 20. Occlusal/Incisal Anatomy (Do not grade for Class III or V) Fossa, grooves, marginal ridges, incisal edge/cusp placement & occlusion |  |  |
| 21. Occlusion |  |  |
| Overall assessment | | |
| 22. Overall ability to complete the procedure |  |  |

List three skills **at least** in the following questions. (Point to the skills using the number it associates with in the above table)

1. What are the trainee’s areas of improvement? ……../……../…….
2. What are the trainee’s areas of excellence? ……../……../…….
3. According to students’ response to i and ii, assess his/her ability to identify areas of improvement or strengths from 0 (points are totally different) to 3 (points are exactly the same).

| Matching in areas of improvement | 0 | 1 | 2 | 3 |
| --- | --- | --- | --- | --- |
| Matching in areas of excellence | 0 | 1 | 2 | 3 |

What is the action plan that you would advise to work on the areas of improvement that you identified in the above question? ..........................................................................................................................................................................................................................................................................................................................................................................................................................................................................................................................................

How long did the feedback session take? ……… minutes Assessor’s signature: ………………………

**DOPS Form for Trainees**

Date: ……/……/2022 Supervisor’s name:. ……….............. Trainee’s name: ………………..
Procedure: Amalgam/Composite/GIC Class: I / II / III / IV / V / 6 Case complexity: Easy/Moderate/Complex
Evaluate your own performance according to the following scale:

| Scale | 1. Clear fail | 2. Borderline fail | 3. Borderline pass | 4. Clear Pass | 0. Unable to comment |
| --- | --- | --- | --- | --- | --- |

| Skill | Score | Note |
| --- | --- | --- |
| Clinical Knowledge and Judgment | | |
| 1. Clinical assessment, diagnosis and treatment plan |  |  |
| 2. Demonstrates understanding of indications, dental materials and used technique |  |  |
| Professionalism, Patient management and Ergonomics | | |
| 3. Obtaining patient consent after explaining the procedure and possible complications |  |  |
| 4. Pre-procedural preparation |  |  |
| 5. Infection control |  |  |
| 6. Pain and anxiety management |  |  |
| 7. Communication skills with patient and team |  |  |
| 8. Patient education |  |  |
| 9. Time management |  |  |
| 10. Ergonomics |  |  |
| Tooth Preparation | | |
| 11. Isolation |  |  |
| 12. Initial and Final access (*over or under-extended, adjacent tooth damage)* |  |  |
| 13. Caries removal |  |  |
| 14. Unsupported enamel removal |  |  |
| Tooth Restoration | | |
| 15. Matrix placement and Wedging |  |  |
| 16. Etching and bonding |  |  |
| 17. Cavosurface (Excess/Submargination) |  |  |
| 18. Restoration Surface/Shade |  |  |
| 19. Axial Anatomy (Facial, lingual, proximal contours) |  |  |
| 20. Occlusal/Incisal Anatomy (Do not grade for Class III or V) Fossa, grooves, marginal ridges, incisal edge/cusp placement & occlusion |  |  |
| 21. Occlusion |  |  |
| Overall assessment | | |
| 22. Overall ability to complete the procedure |  |  |

List three skills **at least** in the following questions. (Point to the skills using the number it associates with in the above table)

1. What are your areas of improvement? ……../……../…….
2. What are your areas of excellence? ……../……../…….

What is the action plan that you would advise to work on the areas of improvement that you identified in the above question? ..........................................................................................................................................................................................................................................................................................................................................................................................................................................................................................................................................

1. Your experience with the current assessment method is positive: Strongly disagree/ Disagree/ Agree/ Strongly agree
2. You benefited from self-evaluating with the supervisor: Strongly disagree/ Disagree/ Agree/ Strongly agree

Trainee’s signature:…………………..
