# Supplementary material for: Evaluation of a workplace assessment method designed to improve self-assessment in operative dentistry: a quasi-experiment
Source: BMC Med Educ. 2023 Jul 3;23:491. doi: 10.1186/s12909-023-04474-z (PMC10318704; doi:10.1186/s12909-023-04474-z)
Supplement: Supplementary file 2 — Supplementary Material 2 [file 12909_2023_4474_MOESM2_ESM.docx]

**English version of the Grading rubric**

| **Item** | **1. Clear fail** | **2. Borderline fail** | **3. Borderline pass** | **4. Clear Pass** |
| --- | --- | --- | --- | --- |
| **General guide for assessment** | The trainee is unable to perform the procedure in an acceptable manner | **The trainee can perform the procedure under direct supervision** | **The trainee can perform the procedure under indirect supervision** | **The trainee can be allowed to perform the procedure indepentently** |
| **Clinical knowledge and judgement** |  |  |  |  |
| **1. Clinical assessment, diagnosis and treatment plan** | Unable to identify/conduct appropriate diagnostic tests | Able to identify/conduct appropriate diagnostic tests  Unable to identify a differential or definitive diagnosis | Requires assistance to establish a differential diagnosis and treatment plan based on the data collected | Requires minimum assistance to establish a differential diagnosis and treatment plan based on the data collected |
| **2. Demonstrates understanding of indications, dental materials and used technique** | No to little knowledge or gives inaccurate or wrong information | Have some knowledge of what needs to be done but missing some or all critical points (requires lots of help) | -Have acceptable knowledge of what needs to be done(requires some help) -Addresses all main steps  -Answers follow up questions fairly with some minor errors | Have a clear picture of what needs to be done (requires minimum help) -Answers follow up questions well and clearly with few errors |
| ***Professionalism, patient management and ergonomics*** |  |  |  |  |
| **3. Obtaining patient consent after explaining the procedure and possible complications** | -Doesn’t explain anything to the patient or explains minimally. | -Explains to the patient partially, but doesn’t give the patient the chance to ask questions-his explanation is not easy to understand by the patient | -Explains the whole procedure fairly, allows the patient to ask questions. -Doesn’t address the patient concerns properly. | -Explains the whole procedure well, allows the patient to ask questions. -Addresses the patient concerns properly. -Fairly aware of these concerns |
| **4. Pre-procedural preparation** | -Unprepared, unaware of the steps and the procedure to satisfactorily meet the required standards | -Minimally prepared -Unclean, Untidy working place | -Acceptable preparation -Clean and tidy working place | -Good preparation  -Clean and tidy working place |
| **5. Infection control** | -Cannot demonstrate any infection control techniques nor safe disposal of sharps  Makes any serious violations of infection control protocols | -Demonstrates some proper infection control technique and safe disposal, but requires lots of assistance and reminders. | -Demonstrates acceptable infection control technique and safe disposal with minimum assistance | -Demonstrates proper infection control and safe disposal of sharps  minimum instruction is needed |
| **6. Pain, anxiety management** | There is no attempt to lessen patients’ anxiety or pain | There is minor attempt to manage anxiety and pain but there is no obvious emphatic attitude or behavior | -Acceptable pain control  -Demonstrates some compassion and respect for patient  -forewarns the patient that a discomfort or pain might follow a certain step of the procedure | -Good pain control  -patient is comfortable and the trainee shows obvious empathetic attitude towards the patient.  -forewarns the patient that a discomfort or pain might follow a certain step of the procedure |
| **7. Communication skills with patient and team** | -ineffective or no communication with the patient and the team  -failure to | -little communication  -doesn’t communicate with the patient during the procedure | -acceptable communication  -shows respect to the patient and team  - seeks feedback from the patient a little bit and shows open-mindedness | -excellent communication skills  -shows respect to the patient and team  -actively seeks feedback from the patient |
| **11. Patient education**  **-medicine, raising awareness**  **-telling the patient when eating is allowed**  **-informing the patient of the possibility of post-procedural sensitivity (gingival or odontogenic)**  **- expected longevity of the restoration**  **-instructions to cleaning and brushing teeth (use of dental floss)** | Not done | Done poorly or inappropriately (missed some essential points) | Done fairly | Done well |
| **Time management** | - Time was improperly managed, not finished on time and/or the patient must return to complete procedure. | -Acceptable standard was not totally met with major assistance Student finishes a bit late with the patient | -Acceptable preparation and time management -Student finishes on time (treatment and paperwork) - no time is spared for cleaning and packing dental tools and materials | -Student is prepared to perform the procedure with minimum assistance   -Student finishes on time (treatment and paperwork). -Have enough time to clean and pack dental tools and materials |
| **Ergonomics** | No skills or knowledge on how to position oneself or the patient | Poor skill (needs instructions) | Acceptable skill (trainee adjusts position repeatedly during procedure) | Good skill (works comfortably and safely generally) |
| **Tooth preparation** |  |  |  |  |
| **11. Isolation** | -Unable to conduct proper isolation technique  -very limited knowledge regarding isolation technique | -needs plenty of directions to maintain appropriate isolation  -has basic knowledge regarding isolation technique | - acceptable isolation with few issues  -acceptable knowledge regarding isolation technique | -good isolation through out the whole procedure  -needs minor directions regarding isolation technique |
| **12. Initial and final access (over-/under-extension/adjacent tooth damage)** | -unable to achieve proper cavity shape without direct supervision  -overextended preparation  -adjacent tooth damage | -acceptable cavity preparation  -minor overextension or under-extension | -good cavity shape  - satisfactory preparation (no under- or over extension) | -Cavity shape and walls are prepared very well with minor or no issues  -achieving minimal preparation principle when possible |
| **13. Caries removal** | -obvious over existence of caries upon initial evaluation  -limited knowledge on caries removal technique | -existence of caries on Dentin-enamel junction  -acceptable knowledge on caries removal technique | -very limited caries existence upon initial evaluation  -good knowledge regarding caries removal technique | -Full caries removal with little over-extension  -very good knowledge regarding caries removal technique |
| **14. Unsupported enamel removal** | -existence of unsupported enamel in an overly fashion upon initial evaluation | -existence of unsupported enamel that can be spotted directly upon initial evaluation | -minor extension of unsupported enamel upon initial evaluation | -no unsupported enamel upon initial evaluation  -well prepared margins |
| **Restoration** |  |  |  |  |
| **15. wedging and matrix placement** | -unable to use wedges and matrix without major assistance  -not using the matrix or wedges in the proper stage  -not conducting pre-wedging | -needs plenty of instructions and directions to use the matrix and wedges | -needs some instructions and directions when using wedges and matrix | -good skill in using wedges and matrix  -able to work semi-independently |
| **16. Etching and bonding (Composite)** | -unable to perform etching and bonding without major assistance  -significant issues during the process  -little knowledge regarding systems of bonding, generations, differences and method of application | Able to perform etching and bonding in an acceptable manner  -needs plenty of instructions and directions  -acceptable knowledge regarding systems of bonding, generations, differences and method of application | -able to perform etching and bonding well  -needs some instructions  -good knowledge regarding systems of bonding, generations, differences and method of application | -able to perform etching and bonding very well and independently  -very good knowledge regarding systems of bonding, generations, differences and method of application |
| **17. Cavosurface (excess/submargination)** | -cracks and fissures in the restoration that exposes the dentin  -clinically unacceptable restoration notches | -v shaped cracks can be probed but without reaching the dentin.  -notches (unacceptable clinically) | -minor local v-shaped cracks  -multiple minor notches | No cracks  Very minor restoration notches |
| **18. Color matching and/or surface polishing** | - color mismatching (clinically unacceptable)  -very rough and cracked restoration | -color mismatching (clinically unacceptable)  -rough texture (clinically unacceptable) | -minor color mismatch (acceptable clinically)  -restoration is rougher than the proximal enamel but it’s clinically acceptable | -very good color matching  -restoration texture is equivalent to that of the proximal |
| **19. Axial anatomy (buccal, lingual, proximal, contact point)** | -over or under convexity/ concavity (clinically unacceptable)  -no contact point with proximals | -over or under convexity/concavity (clinically unacceptable)  -acceptable contact point | --minor over or under convexity/concavity (clinically acceptable)  -good contact point | - convexity appropriate with the tooth shape  -excellent and firm contact point |
| **20. Occlusal/Incisal edge anatomy (not to be evaluated in class III or V) Fossa, grooves, marginal ridges, cusp placement & occlusion (poor)** | -occlusal/incisal anatomical form unacceptable clinically  -not adhering to basic anatomical shape | - acceptable occlusal/incisal form  -basic anatomical forms are maintained | -good occlusal/incisal form  - some asymmetry with proximals (clinically acceptable) | -proper occlusal/incisal form  -similar form with proximals |
| **21. Occlusion** | -restoration is significantly lower or higher than the occlusal plane  -dynamic occlusal is affected | --restoration is lower or higher than the occlusal plane (modifications are required)  -dynamic occlusal is affected | -restoration is slightly lower or higher than the occlusal plane  -dynamic occlusion is not affected | -restoration is appropriate (does not affect the occlusal plane)  -dynamic occlusion has been examined and maintained  -occlusion has been examined and registered before procedure. |
| **22. overall assessment** | The trainee is unable to perform the procedure in an acceptable manner | The trainee can perform the procedure under direct supervision | The trainee can perform the procedure under indirect supervision | The trainee can be allowed to perform the procedure independently |
