# Supplementary material for: Evaluation of a workplace assessment method designed to improve self-assessment in operative dentistry: a quasi-experiment
Source: BMC Med Educ. 2023 Jul 3;23:491. doi: 10.1186/s12909-023-04474-z (PMC10318704; doi:10.1186/s12909-023-04474-z)
Supplement: Supplementary file 3 — Supplementary Material 3 [file 12909_2023_4474_MOESM3_ESM.docx]

**Table S1.** Self-assessment mean scores of each assessment criteria at each of the 5 encounters.

| **Self-assessment: to see if self-assessment changed** | | | | | |
| --- | --- | --- | --- | --- | --- |
|  | **1^st^ encounter** | **2^nd^ encounter** | **3^rd^ encounter** | **4^th^ encounter** | **5^th^ encounter** |
| Criteria | M (90% CI) | M (90% CI) | M (90% CI) | M (90% CI) | M (90% CI) |
| ***Clinical knowledge and judgment*** |  |  |  |  |  |
| 1. Clinical examination, diagnosis and treatment planning | 3.34(3.07-3.61) | 3.21(3.02-3.41) | 3.34(3.14-3.53) | 3.09(2.87-3.31) | 3.06(2.77-3.34) |
| 2. Demonstrates understanding of indications, dental materials and used technique | 3.15(2.92-3.38) | 3.00(2.73-3.26) | 3.34(3.14-3.53) | 3.18(3.01-3.36) | 2.93(2.61-3.25) |
| ***Professionalism, patient management and ergonomics*** |  |  |  |  |  |
| 3. Obtaining patient consent after explaining the procedure and possible complications | 3.46(3.20-3.72) | 3.12(2.84-3.40) | 3.37(3.19-3.55) | 3.28(3.02-3.53) | 3.09(2.75-3.42) |
| 4. Pre-procedural preparation | 3.15(2.98-3.32) | 2.96(2.68-3.24) | 3.18(2.89-3.47) | 3.09(2.88-3.30) | 2.93(2.72-3.15) |
| 5. Infection control | 3.03(2.80-3.25) | 3.09(2.86-3.32) | 2.81(2.54-3.08) | 3.00(2.78-3.21) | 3.00(2.77-3.22) |
| 6. Pain, anxiety management | 3.34(3.10-3.57) | 3.00(2.78-3.21) | 3.40(2.20-3.60) | 3.28(3.06-3.49) | 2.96(2.71-3.22) |
| 7. Communication skills with patient and team | 3.25(2.98-3.51) | 3.18(2.93-3.44) | 3.15(2.95-3.35) | 3.25(3.02-3.47) | 3.12(2.86-3.38) |
| 8. Patient education | 2.87(2.59-3.1) | 2.62(2.24-3.00) | 2.87(2.53-3.21) | 3.03(2.83-3.22) | 2.87(2.56-3.18) |
| 9. Time management | 2.31(2.00-2.62) | 2.81(2.52-3.10) | 2.75(2.43-3.06) | 2.68(2.43-2.94) | 2.65(2.36-2.94) |
| 10. Ergonomics | 2.53(2.25-2.80) | 2.62(2.64-2.88) | 2.59(2.31-2.87) | 2.46(2.21-2.72) | 2.46(2.16-2.77) |
| ***Tooth preparation*** |  |  |  |  |  |
| 11. Isolation | 2.81(2.59-3.03) | 2.71(2.45-2.98) | 3.06(2.82-3.30) | 2.62(2.28-2.96) | 2.53(2.20-2.85) |
| 12. Initial and final access (over-/under-extension/adjacent tooth damage) | 2.96(2.72-3.21) | 3.00(2.75-3.24) | 3.03(2.79-3.26) | 2.90(2.64-3.16) | 2.87(2.69-3.05) |
| 13. Caries removal | 2.53(2.13-2.92) | 2.84(2.51-3.17) | 2.87(2.53-3.21) | 2.75(2.45-3.07) | 2.87(2.58-3.16) |
| 14. Unsupported enamel removal | 3.37(3.17-3.57) | 2.96(2.69-3.23) | 2.87(2.60-3.14) | 2.59(2.30-2.88) | 2.93(2.75-3.12) |
| ***Tooth restoration*** |  |  |  |  |  |
| 15. wedging and matrix placement | 1.84(1.44-2.24) | 1.81(1.38-2.23) | 2.28(1.94-2.61) | 2.00(1.59-2.41) | 2.15(1.69-2.61) |
| 16. Etching and bonding (Composite) | 2.96(2.63-3.30) | 2.84(2.43-3.25) | 3.09(2.78-3.4) | 3.00(2.67-3.32) | 2.40(1.95-2.85) |
| 17. Cavosurface (excess/submargination) | 2.78(2.52-3.04) | 3.00(2.75-3.24) | 3.06(2.83-3.29) | 2.90(2.68-3.12) | 2.96(2.68-3.24) |
| 18. Color matching and/or surface polishing | 2.71(2.45-2.98) | 2.75(2.40-3.09) | 3.09(2.81-3.37) | 2.90(2.63-3.17) | 2.81(2.47-3.14) |
| 19. Axial anatomy (buccal, lingual, proximal, contact point) | 2.53(2.25-2.80) | 2.56(2.25-2.86) | 2.56(2.20-2.91) | 2.50(2.22-2.77) | 2.78(2.50-3.05) |
| 20. Occlusal/Incisal edge anatomy (not to be evaluated in class III or V) | 1.93(1.49-2.38) | 1.62(1.18-2.06) | 2.18(1.74-2.62) | 2.18(1.76-2.60) | 2.43(2.05-2.81) |
| 21. Occlusion | 2.75(2.30-3.19) | 2.59(2.14-3.04) | 2.59(2.19-2.99) | 2.59(2.21-2.97) | 2.53(2.10-2.95) |
| ***22. Overall performance assessment*** | 3.00(2.82-3.17) | 2.75(2.47-3.02) | 2.50(2.10-2.89) | 2.59(2.29-2.89) | 2.65(2.38-2.92) |

M: mean, 90% CI: 9*0*% confidence interval.

**Table S2.**Teachers assessment mean scores of each assessment criteria at each of the 5 encounters

| **Teacher assessment: to see if performance changed** | | | | | |
| --- | --- | --- | --- | --- | --- |
|  | **1^st^ encounter** | **2^nd^ encounter** | **3^rd^ encounter** | **4^th^ encounter** | **5^th^ encounter** |
| Criteria | M (90% CI) | M (90% CI) | M (90% CI) | M (90% CI) | M (90% CI) |
| ***Clinical knowledge and judgment*** |  |  |  |  |  |
| 1. Clinical examination, diagnosis and treatment planning | 2.53(2.28-2.79) | 2.73(2.48-2.97) | 2.64(2.44-2.84) | 2.56(2.29-2.83) | 2.65(2.41-2.89) |
| 2. Demonstrates understanding of indications, dental materials and used technique | 2.42(2.14-2.71) | 2.89(2.67-3.11) | 2.67(2.44-2.90) | 2.73(2.44-3.02) | 2.33(2.03-2.63) |
| ***Professionalism, patient management and ergonomics*** |  |  |  |  |  |
| 3. Obtaining patient consent after explaining the procedure and possible complications | 2.03(1.81-2.25) | 2.55(2.23-2.87) | 2.38(2.06-2.70) | 2.61(2.21-3.01) | 2.42(2.08-2.75) |
| 4. Pre-procedural preparation | 2.56(2.31-2.81) | 2.74(2.50-2.97) | 2.61(2.34-2.88) | 2.71(2.42-3.01) | 2.51(2.25-2.77) |
| 5. Infection control | 2.37(2.10-2.64) | 2.78(2.55-3.00) | 2.67(2.41-2.94) | 2.62(2.33-2.91) | 2.59(2.34-2.84) |
| 6. Pain, anxiety management | 2.40(2.14-2.65) | 2.53(2.26-2.80) | 2.56(2.32-2.80) | 2.86(2.53-3.20) | 2.50(2.19-2.80) |
| 7. Communication skills with patient and team | 2.09(1.84-2.33) | 2.63(2.39-2.87) | 2.74(2.46-3.01) | 2.80(2.48-3.11) | 2.59(2.31-2.86) |
| 8. Patient education | 2.50(2.16-2.83) | 2.58(2.31-2.85) | 2.24(1.93-2.54) | 2.78(2.41-3.15) | 2.47(2.19-2.75) |
| 9. Time management | 2.03(1.76-2.29) | 2.65(2.31-2.99) | 2.32(2.09-2.55) | 2.65(2.33-2.98) | 2.58(2.32-2.83) |
| 10. Ergonomics | 2.00(1.79-2.20) | 2.51(2.25-2.77) | 2.62(2.42-2.82) | 2.30(2.00-2.59) | 2.13(1.8-2.38) |
| ***Tooth preparation*** |  |  |  |  |  |
| 11. Isolation | 2.46(2.19-2.73) | 2.58(2.21-2.95) | 2.60(2.28-2.91) | 2.76(2.39-3.14) | 2.93(2.63-3.23) |
| 12. Initial and final access (over-/under-extension/adjacent tooth damage) | 2.48(2.18-2.77) | 2.83(2.55-3.11) | 2.60(2.32-2.87) | 2.93(2.56-3.29) | 2.81(2.50-3.12) |
| 13. Caries removal | 1.96(1.62-2.29) | 2.56(2.23-2.89) | 2.33(1.96-2.70) | 2.68(2.22-3.15) | 2.78(2.43-3.14) |
| 14. Unsupported enamel removal | 3.07(2.76-3.37) | 2.96(2.65-3.27) | 2.87(2.52-3.22) | 2.96(2.51-3.40) | 3.04(2.69-2.39) |
| ***Tooth restoration*** |  |  |  |  |  |
| 15. wedging and matrix placement | 2.12(1.86-2.38) | 2.56(2.19-2.93) | 2.56(2.20-2.91) | 2.79(2.37-3.20) | 2.95(2.60-3.29) |
| 16. Etching and bonding (Composite) | 2.78(2.56-3.00) | 3.00(2.71-3.28) | 2.84(2.54-3.13) | 3.24(2.87-3.60) | 3.38(3.04-3.73) |
| 17. Cavosurface (excess/submargination) | 2.29(2.03-2.54) | 2.87(2.60-3.14) | 2.87(2.62-3.12) | 2.87((2.58-3.16) | 2.93(2.66-3.20) |
| 18. Color matching and/or surface polishing | 3.00(2.76-3.23) | 3.06(2.83-3.29) | 3.06(2.85-3.28) | 3.25(2.94-3.55) | 3.40(3.14-3.66) |
| 19. Axial anatomy (buccal, lingual, proximal, contact point) | 2.25(1.94-2.56) | 2.67(2.33-3.01) | 3.00(2.74-3.25) | 2.48(2.12-2.84) | 2.86(2.57-3.15) |
| 20. Occlusal/Incisal edge anatomy (not to be evaluated in class III or V) | 2.58(2.25-2.90) | 2.86(2.51-3.20) | 3.20(2.88-3.51) | 2.84(2.41-3.26) | 3.11(2.79-3.43) |
| 21. Occlusion | 2.42(2.09-2.73) | 2.90(2.49-3.31) | 2.95(2.67-3.24) | 3.00(2.68-3.31) | 3.05(2.67-3.44) |
| ***22. Overall performance assessment*** | 2.22(1.98-2.47) | 2.43(2.22-2.65) | 2.25(2.02-2.47) | 2.45(2.09-2.80) | 2.45(2.21-2.68) |

M: mean, 90% CI: 9*0*% confidence interval.

**Table S3.** Repeated measure ANOVA for self-assessment at each assessment criteria in the five assessment encounters.

| **Self-assessment: to see if self-assessment changed** | | | | | |
| --- | --- | --- | --- | --- | --- |
|  | **Repeated measures ANOVA^2^** | | | |  |
| Criteria | *F* | *P* | Partial Eta squared | Observed power |  |
| ***Clinical knowledge and judgment*** |  |  |  |  |  |
| 1. Clinical examination, diagnosis and treatment planning | 1.080 | 0.370 | 0.034 | 0.459 |  |
| 2. Demonstrates understanding of indications, dental materials and used technique | 1.360 | 0.252 | 0.042 | 0.490 |  |
| ***Professionalism, patient management and ergonomics*** |  |  |  |  |  |
| 3. Obtaining patient consent after explaining the procedure and possible complications | 1.209 | 0.311 | 0.038 | 0.500 |  |
| 4. Pre-procedural preparation | 0.602 | 0.444 | 0.019 | 0.196 |  |
| 5. Infection control | 0.814 | 0.518 | 0.026 | 0.372 |  |
| 6. Pain, anxiety management | 2.756 | 0.031 | 0.082 | 0.838 |  |
| 7. Communication skills with patient and team | 0.187 | 0.945 | 0.006 | 0.159 |  |
| 8. Patient education | 0.946 | 0.440 | 0.030 | 0.416 |  |
| 9. Time management | 1.535 | 0.196 | 0.047 | 0.594 |  |
| 10. Ergonomics | 0.381 | 0.822 | 0.012 | 0.223 |  |
| ***Tooth preparation*** |  |  |  |  |  |
| 11. Isolation | 1.616 | 0.174 | 0.050 | 0.616 |  |
| 12. Initial and final access (over-/under-extension/adjacent tooth damage) | 0.261 | 0.902 | 0.008 | 0.183 |  |
| 13. Caries removal | 0.504 | 0.711 | 0.016 | 0.266 |  |
| 14. Unsupported enamel removal | 4.373 | 0.002 | 0.124 | 0.962 |  |
| ***Tooth restoration*** |  |  |  |  |  |
| 15. wedging and matrix placement | 0.722 | 0.578 | 0.023 | 0.341 |  |
| 16. Etching and bonding (Composite) | 1.492 | 0.209 | 0.046 | 0.582 |  |
| 17. Cavosurface (excess/submargination) | 0.589 | 0.671 | 0.019 | 0.295 |  |
| 18. Color matching and/or surface polishing | 0.748 | 0.561 | 0.024 | 0.350 |  |
| 19. Axial anatomy (buccal, lingual, proximal, contact point) | 0.459 | 0.766 | 0.015 | 0.250 |  |
| 20. Occlusal/Incisal edge anatomy (not to be evaluated in class III or V) | 1.669 | 0.161 | 0.051 | 0.629 |  |
| 21. Occlusion | 0.114 | 0.977 | 0.004 | 0.135 |  |
| ***22. Overall performance assessment*** | 1.367 | 0.249 | 0.042 | 0.547 |  |

9*0*% confidence interval. Sphericity assumed.

**Table S4.** Repeated measure ANOVA for teacher assessment at each assessment criteria in the five assessment encounters.

| **Teacher assessment** | | | | | |
| --- | --- | --- | --- | --- | --- |
|  | **Repeated measures ANOVA^2^** | | | |  |
| Criteria | *F* | *P* | Partial Eta squared | Observed power |  |
| ***Clinical knowledge and judgment*** |  |  |  |  |  |
| 1. Clinical examination, diagnosis and treatment planning | 0.254 | 0.906 | 0.013 | 0.179 |  |
| 2. Demonstrates understanding of indications, dental materials and used technique | 2.271 | 0.070 | 0.118 | 0.751 |  |
| ***Professionalism, patient management and ergonomics*** |  |  |  |  |  |
| 3. Obtaining patient consent after explaining the procedure and possible complications | 0.465 | 0.761 | 0.037 | 0.245 |  |
| 4. Pre-procedural preparation | 0.573 | 0.683 | 0.020 | 0.289 |  |
| 5. Infection control | 1.294 | 0.276 | 0.041 | 0.525 |  |
| 6. Pain, anxiety management | 0.570 | 0.685 | 0.028 | 0.285 |  |
| 7. Communication skills with patient and team | 4.199 | 0.003 | 0.144 | 0.954 |  |
| 8. Patient education | 1.760 | 0.186 | 0.306 | 0.567 |  |
| 9. Time management | 2.524 | 0.045 | 0.080 | 0.803 |  |
| 10. Ergonomics | 3.068 | 0.020 | 0.106 | 0.873 |  |
| ***Tooth preparation*** |  |  |  |  |  |
| 11. Isolation | 0.857 | 0.493 | 0.038 | 0.383 |  |
| 12. Initial and final access (over-/under-extension/adjacent tooth damage) | 0.231 | 0.920 | 0.016 | 0.171 |  |
| 13. Caries removal | 4.926 | 0.002 | 0.309 | 0.971 |  |
| 14. Unsupported enamel removal | 0.386 | 0.817 | 0.046 | 0.215 |  |
| ***Tooth restoration*** |  |  |  |  |  |
| 15. wedging and matrix placement | 1.000 | 0.422 | 0.111 | 0.406 |  |
| 16. Etching and bonding (Composite) | 0.967 | 0.441 | 0.121 | 0.391 |  |
| 17. Cavosurface (excess/submargination) | 2.621 | 0.038 | 0.083 | 0.818 |  |
| 18. Color matching and/or surface polishing | 1.191 | 0.321 | 0.054 | 0.489 |  |
| 19. Axial anatomy (buccal, lingual, proximal, contact point) | 2.564 | 0.047 | 0.138 | 0.799 |  |
| 20. Occlusal/Incisal edge anatomy (not to be evaluated in class III or V) | 0.580 | 0.681 | 0.104 | 0.263 |  |
| 21. Occlusion | 3.217 | 0.075 | 0.617 | 0.726 |  |
| ***22. Overall performance assessment*** | 0.755 | 0.557 | 0.026 | 0.351 |  |

9*0*% confidence interval. Sphericity assumed.
